# Editorial: Understanding anaesthetic effects on aquatic animals

**DOI:** 10.3389/fvets.2025.1734380

**Published:** 2025-11-20

**Authors:** Luís Félix, Ana M. Valentim, Carlos Venâncio

**Affiliations:** 1 Centre for the Research and Technology of Agroenvironmental and Biological Sciences, CITAB, Inov4Agro, Universidadede Trás-os-Montes e Alto Douro, UTAD, Vila Real, Portugal; 2 i3S - Instituto de Investigação e Inovação em Saúde, Universidade do Porto, Porto, Portugal; 3 Animal and Veterinary Research Centre (CECAV), UTAD, Vila Real, Portugal; 4 Animal Science Department, School of Agrarian and Veterinary Sciences (ECAV), UTAD, Vila Real, Portugal

**Keywords:** anesthesia, welfare, machine learning, decision-making models, nanoemulsions, aquatic models

Whether in aquaculture or laboratory settings, the need to restrain, transport, or surgically manipulate aquatic species makes anesthetic protocols indispensable. Anesthetics are physical or pharmacological agents that induce a state of unconsciousness allowing researchers and aquaculture practitioners to perform procedures with minimal stress, pain, and physiological disturbance to the animal. Anesthetic agents act by depressing the central nervous system, resulting in a graded series of responses that include loss of equilibrium, reduced motor activity, and ultimately loss of consciousness. Effective anesthesia ensures not only the welfare of the organism but also the reliability of experimental results and the safety of personnel handling the animals (1). For decades, compounds such as tricaine methanesulfonate (MS-222) and clove oil derivatives have dominated aquatic anesthesia (2). These agents are effective but recent reports have highlighted concerns about residues, species-specific responses, variable regulation across countries, and welfare implications at higher concentrations, which have prompted the search for alternatives (3, 4). Considering the increasing importance of anesthetic use in aquaculture and experimental research, this Research Topic of Frontiers in Veterinary Science focused on advancing the understanding of anesthetic agents and their physiological effects in aquatic organisms.

Through approaches encompassing the use and refinement of natural anesthetic formulation, the development of predictive machine learning models, and the integration of multi-criteria decision-making frameworks, this Research Topic offered novel insights into the safe and sustainable application of anesthetic protocols in aquatic sciences. By addressing the poor water solubility and volatility of essential oils, and the risk of using high ethanol concentrations as solvent, Zeng et al. developed nanoemulsions and self-microemulsifying drug delivery systems for *Magnolia denudata* essential oil to be applied to juvenile *Lateolabrax maculatus*. This study highlighted how modern delivery systems can improve both the performance and safety of essential oil-based anesthetics for fish. In addition of causing a quick anesthetic induction, the prepared formulations exhibited a safe histopathological and biochemical profile with minimal adverse effects on fish health and behavior, suggesting their potential for safe application in aquaculture and research settings. While future integration of mechanistic pharmacokinetic data, environmental safety evaluation and cost-benefit analyses are required, this study highlighted the potential of nanotechnology-enabled anesthesia in sustainable aquaculture and aquatic research.

The study by Minaz focused on the use of nutmeg (*Myristica fragrans*) essential oil as a sedative and anesthetic agent in Common carp (*Cyprinus carpio*). At lower concentrations, nutmeg oil effectively induced sedation and anesthesia within clinically acceptable timeframes, with transient physiological disturbances that largely resolved within several hours. In contrast, higher concentrations were linked to prolonged recovery times, histopathological alterations in gill tissue, and biomarkers indicative of DNA damage. The optimal concentration was achieved using a multi-criteria decision making called PROMETHEE (Preference Ranking Organization Method for Enrichment) where 15 evaluation criteria related to induction and recovery times, cost analysis, hematological parameters, histological changes, and DNA comet assay were identified. While demonstrating the value of a broader suite of endpoints, the results underscore the critical principle that “natural” does not inherently mean “safe”. Rather, the authors highlighted the necessity of rigorous concentration–response evaluations to identify concentrations that optimize anesthetic efficacy while safeguarding animal welfare and minimizing sublethal toxicological effects. The PROMETHEE multi-criteria decision model was further developed for different species in another study by Minaz et al.. This time, the effect of a commercial formulation composed of an anesthetic mixture of herbal extracts (VetiVital AquaSED includes eugenol, linalool, linalyl acetate, etc.) in Common carp (*Cyprinus carpio*), Rainbow trout (*Oncorhynchus mykiss*), and Danube sturgeon (*Acipenser gueldenstaedtii*) were studied. To evaluate the best anesthetic concentration on each species, PROMETHEE model included 10 criteria related to induction and recovery times, cost analysis, hematological parameters, and histological alterations. This approach goes beyond reporting induction and recovery times by integrating multiple, and often conflicting, criteria, and demonstrated to be applicable to different species. By ranking anesthetic concentrations across this diverse set of measures, the model helped to identify where efficacy and welfare were jointly optimized. Overall, this constituted one of the most innovative contributions in this Research Topic.

Minaz et al. finalized this Research Topic by applying machine learning, specifically artificial neural networks, to predict anesthetic outcomes from nutmeg oil (*Myristica fragrans*) in three freshwater fish species—*Cyprinus carpio* (Common carp), *Acipenser gueldenstaedtii* (Danube sturgeon), and *Oncorhynchus mykiss* (Rainbow trout). By training models on experimental data across the different species, the authors demonstrated that induction and recovery times, as well as hematological responses, can be predicted with high accuracy. The most accurate models were obtained to white blood cell count in all species, whereas induction and recovery times were more species-specific, and these physiological differences were demonstrated in the models. These tools hold enormous promise as they can guide concentration selection, reduce the number of animals needed for pilot testing, and help anticipate physiological stress under different conditions. The implications extend beyond efficiency as predictive models can accelerate the tailoring of protocols to species with limited existing data. They can also incorporate environmental variables—temperature, dissolved oxygen, water chemistry—that shape anesthetic response in real-world conditions.

Despite the progress reflected in these studies, several challenges remain as the choice of anesthetic depends on multiple factors such as species, size, water temperature, salinity, duration and type of the procedure, and the specific physiological parameters under investigation. Long-term effects of anesthesia are still underexplored and regulatory frameworks for natural anesthetics lag behind their growing use, raising questions about standardization, residue safety, and approval processes. Still, the manuscripts gathered in this Research Topic advance the understanding of aquatic anesthesia proposing innovative approaches. The participating authors demonstrate that natural products like *Magnolia denudata* essential oil can be administered more efficiently and safely using novel nanoemulsion-based formulations, and that nutmeg oil shows potential but requires further rigorous evaluation. They show that welfare-oriented endpoints beyond induction and recovery behavior are essential for evaluating anesthetic safety and introduce decision-making models and machine learning as powerful tools to navigate the complexity of protocol optimization. Whether in aquaculture or laboratory science, the safe and effective anesthesia of aquatic animals underpins both scientific progress and ethical responsibility. By embracing the approaches highlighted in this Research Topic, a critical step toward this dual goal is attained while future refinements, including the integration of long-term outcomes and species-specific welfare indicators, will increase the power of such approaches.
